# Da Vinci single-port robotic system current application and future perspective in general surgery: A scoping review

**DOI:** 10.1007/s00464-024-11126-w

**Published:** 2024-08-07

**Authors:** Francesco Celotto, Niccolò Ramacciotti, Alberto Mangano, Giacomo Danieli, Federico Pinto, Paula Lopez, Alvaro Ducas, Jessica Cassiani, Luca Morelli, Gaya Spolverato, Francesco Maria Bianco

**Affiliations:** 1 Department of Surgical, Oncological and Gastroenterological Sciences, University of Padova, Padova, Italy; 2 Department of Translational Research and New Technologies in Medicine and Surgery, University of Pisa, Pisa, Italy; 3 Division of General, Minimally Invasive, and Robotic Surgery, Department of Surgery, University of Illinois at Chicago, Chicago, IL USA; 4 Unit of Biostatistics, Epidemiology and Public Health (UBEP), Department of Cardio-Thoraco-Vascular Sciences and Public Health, University of Padua, Padova, Italy

**Keywords:** Single port, Robotic, General surgery

## Abstract

**Background:**

The da Vinci Single-Port Robot System (DVSP) allows three robotic instruments and an articulated scope to be inserted through a single small incision. It received FDA approval in 2014 and was first introduced in 2018. The aim of this new system was to overcome the limitations of single-incision laparoscopic and robotic surgery. Since then, it has been approved for use only for urologic and transoral surgeries in some countries. It has been used as part of experimental protocols in general surgery.

**Objective:**

By obtaining the CE mark at the end of January 2024, DVSP will soon enter the European market. This review aims to comprehensively describe the applications of DVSP in general surgery.

**Design:**

A search of PubMed, Embase, and Ebsco databases up to March 2024 was conducted, with registration in PROSPERO (CRD42024536430), following the preferred reporting items for Systematic reviews and Meta-analyses for scoping review (PRISMA-Scr) guidelines. All the studies about the use of DVSP in general surgery were included.

**Results:**

Fifty-six studies were included. The following surgical areas of use were identified: transabdominal and transanal colorectal, cholecystectomy, abdominal wall repair, upper gastroesophageal tract, liver, pancreas, breast, and thyroid surgery. The reported surgical and short-term outcomes are promising; a wide range of procedures have been performed safely. Some groups have found advantages, such as faster discharge, shorter operative time, and less postoperative pain compared to multiport robotic surgery.

**Conclusion:**

Five years after its initial clinical applications, the use of the DVSP in general surgery procedures has demonstrated feasibility and safety. Hernia repair, cholecystectomy, and colorectal surgery emerge as the most frequently conducted interventions with this robotic system. Nevertheless, there is anticipation for further studies with larger sample sizes and extended follow-up periods to provide more comprehensive insights and data on the long-term outcomes, including the incidence of incisional hernia.

**Graphical Abstract:**

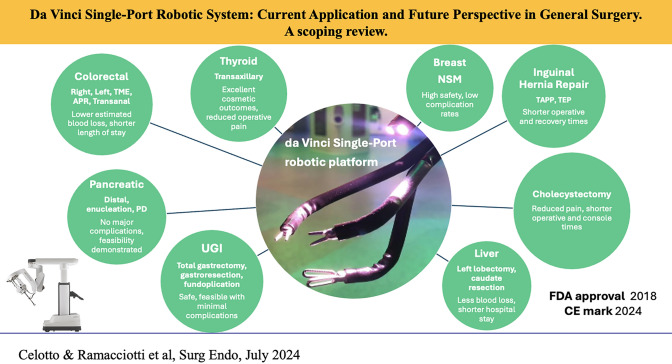

**Supplementary Information:**

The online version contains supplementary material available at 10.1007/s00464-024-11126-w.

Since its introduction in 1985, minimally invasive surgery (MIS) has revolutionized surgical practice. Nowadays, MIS is a safe and effective surgical approach for several diseases, both benign and malignant, and in many cases, it is the standard of care [1–5].

With the goal of making surgery even less invasive, single-incision laparoscopic surgery (SILS) was introduced in the early 1990s. The first intervention performed in general surgery was an appendectomy. SILS is considered an effective modality in terms of postoperative pain management, patient satisfaction, and cosmetic outcomes, but due to technical limitations, such as a reduced ability to triangulate, external and internal clashing, and overall ergonomic discomfort, widespread adoption has been limited and use has been sporadic [3, 4, 6–10]. In numerous instances, apprehension has been expressed regarding the dimensions of the incision, which is susceptible to a higher incidence of incisional hernia development [11, 12].

In the late 1990s, the multiport robot-assisted surgery made its appearance with the first prototypes.

Since then, several platforms for robot-assisted surgery have been developed over time. Robot-assisted surgery is deemed to be safe and effective for many procedures, with the well-known and well-described advantages when compared to laparoscopy [5].

In 2011, Intuitive introduced specialized instruments for the da Vinci Single Site (Intuitive Surgical, Sunnyvale, CA) to perform robotic‐assisted single‐incision procedures with the multiport platforms. Although these innovations reduced [13] some of the technical issues with SILS, the lack of Endowrist, the restricted movement range, and its limited strength prevented its widespread adoption.

The da Vinci Single-Port (DVSP) Surgical System (Intuitive Surgical, Sunnyvale, CA) received Food and Drug Administration (FDA) approval in 2014 and as of 2018 (FDA: K173906) it is approved in the USA for use in transoral and urologic surgery. In urological surgery, it has been widely investigated and adopted with outcomes that are comparable to multiport robotic surgery [14]. For abdominal surgery, it is being used experimentally in some centers in the USA and Asia.

With its single-port architecture and advanced instrument design, including wristed and elbowed instruments and a 3D-HD articulating scope, DVSP enables enhanced maneuverability, dexterity, and visualization within the surgical field overcoming the problem of working in narrow spaces with a single incision of about 25 mm. The docking in DVSP is significantly faster than the da Vinci Xi and the learning curve to master it is shorter than the Si/Xi system, probably due to the presence of a single arm in comparison with the multiport robotic systems [15, 16].

At the end of January 2024 DVSP has received CE mark approval for use in Europe for endoscopic abdominopelvic, thoracoscopic, transoral otolaryngology, transanal colorectal, and breast surgical procedures.

With the advent of this promising new technology, we performed a review of the literature on how the DVSP has been used in general surgery, which has been experimental to date, in order to define the extent to which this new platform can be used and the related outcomes.

## Materials and methods

### Da Vinci single-port basics

In 2018, Intuitive launched on a marked special platform for single incision surgery, the DVSP. The system features a complete redesign of the surgical platform and the instrument arms architecture.

The system is composed by the surgeon console, patient side cart, and the vision cart. The console is similar to the da Vinci multiport Xi, from which it differs for the presence of a virtual navigator that shows the position of robotic instruments and camera in real time. The vision cart maintains the same design as the previous system.

**Fig. 1:**
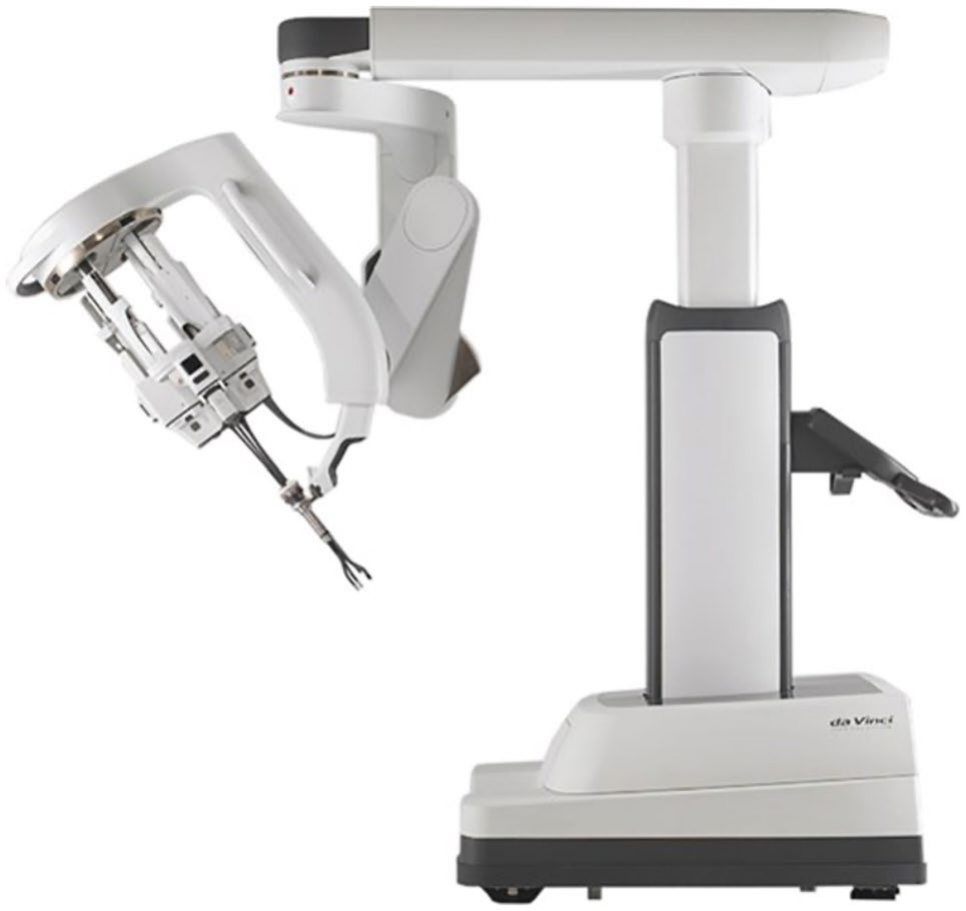
Da Vinci single-port robotic cart

**Fig. 2:**
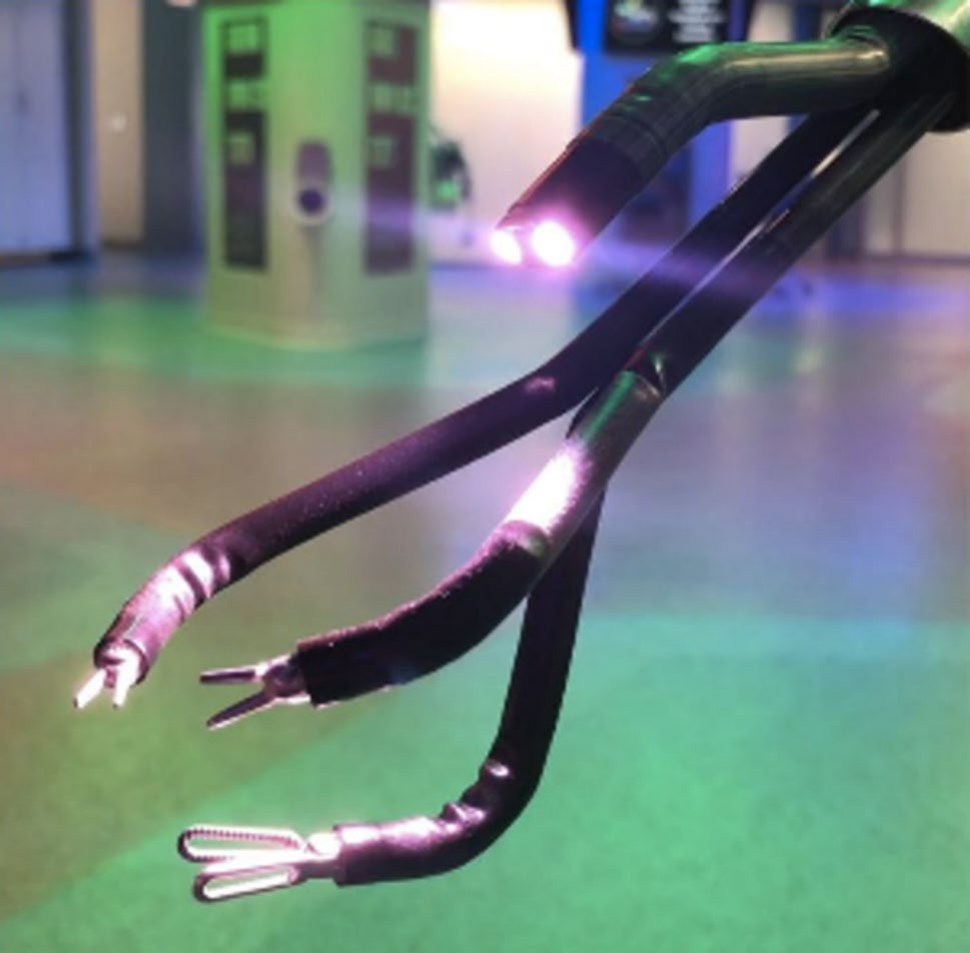
Multijointed endowristed instrument and scope

While keeping the same overhead design, the side cart has a single arm (Fig. 1) and a drive that hosts 3 robotic multijointed, endowristed instruments and an 3D-High-Definition endoscope that is also, for the first time, multijointed (Fig. 2).

**Fig. 3:**
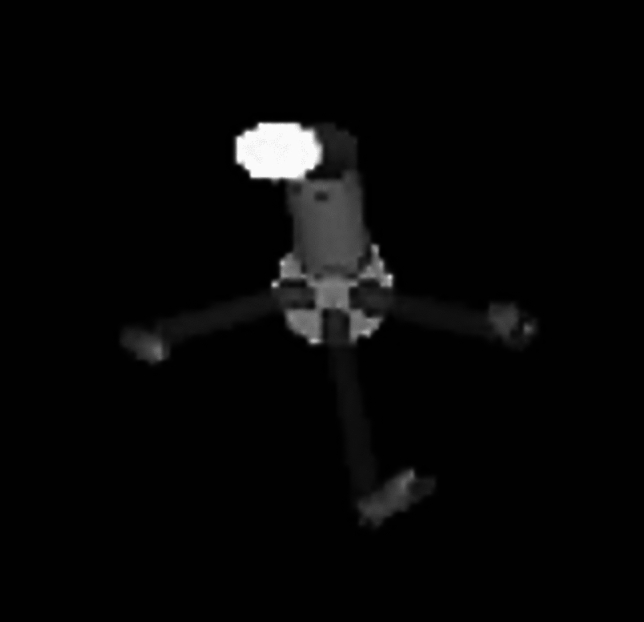
Holographic navigator

By housing the camera and three instruments, grouped together, into a single 25-mm shaft, the collisions are kept to a minimum. The elbow deployment allows for the instruments to deploy in a diamond fashion. The entire operative field can move as a single unit based on the fulcrum of entry at the abdominal wall. In addition, a holographic display on the operative panel (Fig. 3) allows the surgeon to track the orientation of the instruments in reference to themselves internally, to minimize conflicts. The space within the abdominal cavity allows easy deployment of the single-port elbowed instruments which needs a clearance of approximately 10 cm from the entry point [17]. Moreover, thanks to the possibility for the platform’s boom to rotate 360° within and around the port’s remote center, it is suitable for multi-quadrant surgery without the need for re-docking. The rotation movements are controlled by the surgeon at the console or can be completed at bedside [17, 18].

Currently, the instruments available for the DVSP include a Cadiere forceps, round tooth retractor, Maryland and fenestrated bipolar forceps, scissors, spatula and hook monopolar cautery instruments, needle driver, and clip applier. At this time, the system lacks a vessel sealer, stapler, and suction-irrigation system [14, 17].

### Methods

This study was conducted in accordance with the Preferred Reporting Items for systematic reviews and Meta-analysis extension for scoping Reviews (PRISMA-ScR) guidelines [19]. The study protocol has been registered on the International Prospective Register of Systematic Reviews (PROSPERO, registration number CRD42024536430) on April 26, 2024.

### Research question

The DVSP is being used in abdominal surgery under experimental protocol in some centers in the USA and Asia.

The following question led this review: “What have been the applications of the da Vinci Single-Port robotic System in general surgery to date?”.

### Eligibility criteria

Publications related to general surgery using the DVSP that were available in the current literature were included.

The following study designs were considered: randomized controlled trials, controlled clinical trials, observational studies (retrospective and prospective), cohort studies, population-based studies, cross-sectional studies, case–control studies, and case reports.

Exclusion criteria include the use of the Single-Port robot in areas other than general surgery (e.g., gynecology, urology, head and neck, cardiothoracic), use of a multiport robot or other mini-invasive techniques, publications in languages other than English, and publications that are not available in full text.

### Search strategy, study selection, and data collection

A review of the published literature from 2018 until 15 March 2024 was performed in the following databases: PubMed, Embase, and Ebsco. The keywords (including synonyms or equivalent terms) used included “single port,” “SP,” “robotic,” “robotic surgery,” and “surgery”, in combination with Boolean operators (AND, OR). The final search strategy for PUBMED can be found in Additional file 1.

Articles were screened according to the previously described inclusion criteria, and two reviewers independently screened the literature according to the predefined strategy described above. Rayyan web application was used to remove duplicates [20]. Two reviewers (F.C. and N.R.) independently screened the titles and abstracts and cross-checked the results of the studies. Disagreements were resolved by a third reviewer (F.M.B.). Each reviewer extracted the following data variables: title, abstract, and reference details (first author, journal, year). During the review process, both reviewers independently recorded data in separate databases. A comparison was conducted at the end to mitigate selection bias. Additionally, selected manuscripts were grouped according to the surgical specialty of interest.

**Fig. 4:**
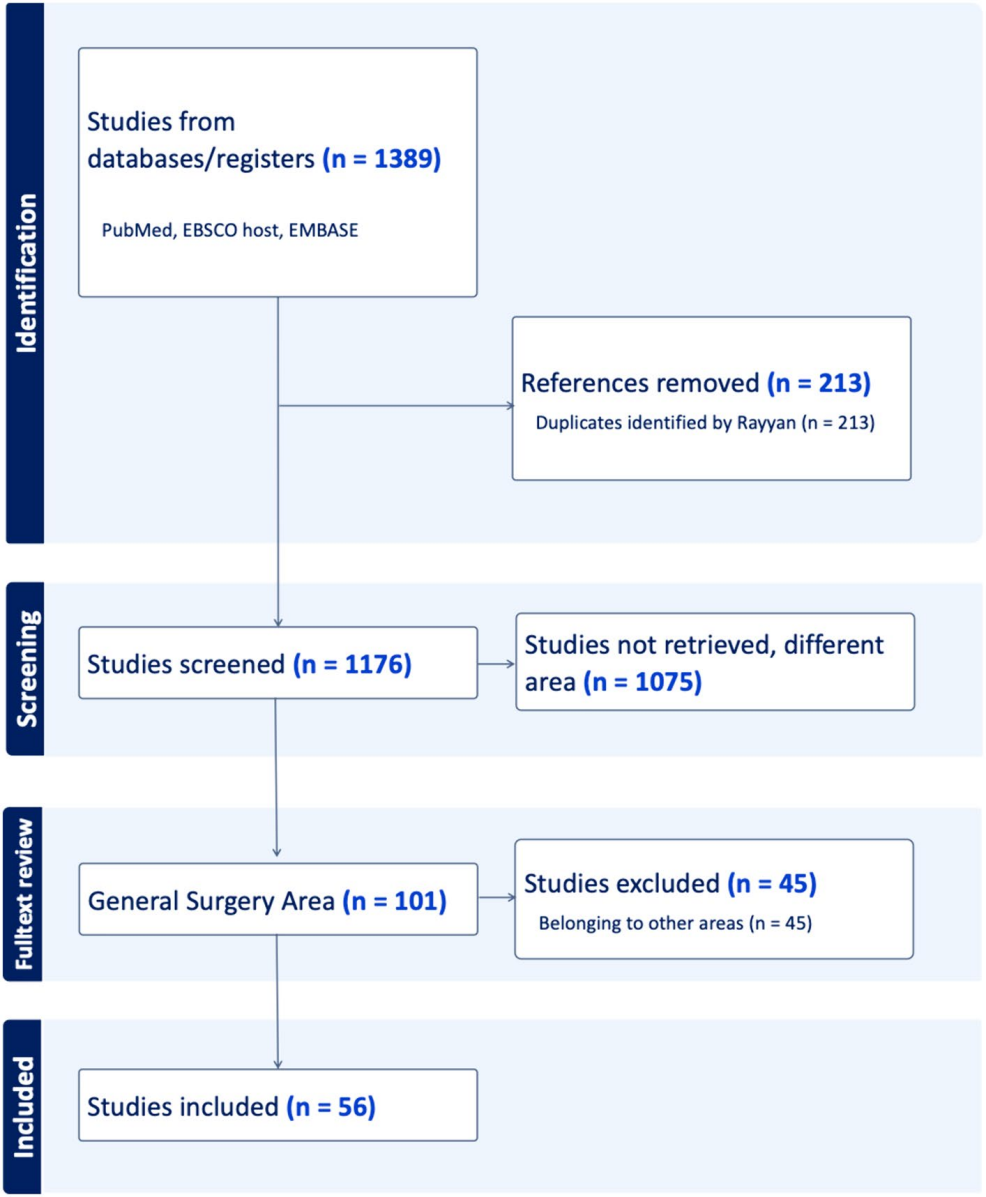
Overview of study selection and PRISMA flowchart

An overview of the study’s methodology is provided in Fig. 4 (PRISMA flowchart).

## Results

The literature search yielded 1389 publications, which were evaluated by two reviewers after removal of duplicates. After abstract screening and full-text reading, 56 papers were included in the review. They were divided into groups based on the pathology treated with DVSP. Twenty-two deal with colorectal surgery, 8 with cholecystectomy, 4 with pancreatic surgery, 3 with upper gastrointestinal (UGI) surgery, 3 with abdominal wall surgery, 3 with liver surgery, 3 with breast surgery, and 8 with thyroid surgery.

### Colorectal surgery

Starting from 2008 surgeons were experimenting single-site laparoscopic and robotic surgery. However, these were limited to few case series due to the well-described technical ergonomic problems of the platform. Although these difficulties, a systematic review showed for single-site surgery lower Estimated Blood Loss (EBL), conversion rate, postoperative pain, and morbidity rate [21, 22].

Eighteen papers reporting on transabdominal colorectal surgery using DVSP were identified (Table 1).

**Table 1:** Colorectal transabdominal experience with da Vinci single-port robotic system

Author	Year	Procedure	Access	Intraop. complications	EBL (ml)	Post-op. compl. (C–D)	Median docking time (min)	Median operative time (min)	Positive margins (%)	LOS
Marks et al. [34]	2020	2 LC	Right lower quadrant	No	40	No	13	306	0	2.5
Marks et al. [35]	2020	1 RC	Left peri-umbilical area	No	100	No	19	219	0	3
Noh et al. [36]	2020	1 AR	Umbilical	No	50	1 (2)	40	255	0	14
		1 LAR	Umbilical	No	50	0	33	300	0	9
		5 RC	Umbilical	No	60	1 (2)	19.8	303	0	7
Kim et al. [25]	2021	5 Rectal resection	Right lower quadrant	1	15	No	4.20	195	0	7
Salem et al. [26]	2021	4 LC	Right lower quadrant	No	91	No	8.4	310	-	2.7
Kim et al. [37]	2021	1 LAR + LPND	Right lower quadrant	No	15	No	3.30	255	0	-
Song et al. [27]	2021	5 RC	Pfannenstiel	No	18	1 (2)	4.40	160	0	7
Piozzi et al. [38]	2022	7 ISR	Right lower quadrant	No	< 50	3 (< 3b)	7	280	0	7
		5 RC	Pfannenstiel			1 (2)	5	220	0	5
		1 TC	Pfannenstiel			0	5	220	0	5
Choo et al. [28]	2022	1 RC	Suprapubic	No	–	No	–	–	–	–
Piozzi et al. [39]	2022	1 TC	Pfannenstiel	No	< 50	No	6	225	0	6
Alshalawi et al. [31]	2023	28 AR	Right lower quadrant	No	20	5 (< 3)	4	184	3.57 (1)	5
Picciariello et al. [18]	2023	2 APR	Left lower quadrant	No	17.5	No	4.2	165	0	5
Jeong et al. [30]	2023	13 LAR/ISR	Right lower quadrant	No	20	1 (3b)	–	180	7.7 (1)	7
Marks et al. [40]	2023	93 colic resection	Transabdominal	2	50	14 (1 > 3a)	5.6	357.15	0	4
Kim et al. [41]	2023	50 Colic resection	Umbilical	0	50	5 (< 3)	14	262.5	0	7
Bae et al. [42]	2024	1 LAR	Umbilical	No	< 50	No	–	160	0	8
Kim et al. [32]	2024	TME	Right lower quadrant	No	27.3	5 (–)	–	160	0	6.2
Keller et al. [29]	2024	6 RC	–	No	150	5 (–)	–	318	1 (2.6)	4
		5 LC								
		32 TATA								
		6 LAR								
		1 APR								

*EBL* estimated blood loss, *LOS* length of stay, *AR* anterior resection, *LAR* low anterior resection, *LAR + LPND* low anterior resection + lateral pelvic node dissection, *RC* right colectomy, *LC* left colectomy, *TC* transverscolectomy, *APR* abdominoperineal resection, *ISR* intersphinteric resection, *TME* total mesorectal excision, *TATA* transanal transabdominal radical proctosigmoidectomy with coloanal anastomosis

The first cases of human transabdominal colorectal surgery using DVSP are reported in the literature by Marks et al. and Kim HJ et al. in the early 2020. They performed two left and one right hemicolectomy and 5 Low Anterior Resection (LAR) for rectal cancer, respectively. No intraoperative complications occurred and a regular postoperative course was reported. This demonstrated the feasibility of the SP approach to colorectal surgery. The technique used in previously reported cases consisted of a horizontal pararectal approach and placement of the 25-mm port through a multiport access platform (e.g., GelPoint or Uniport). This allowed the assistant to use tools, such as suction, staplers, or energy devices [23–25].

Then, case series have been reported with progressively larger sample size, confirming the possibility of performing major colorectal surgery using DVSP with multiple approaches [5, 26, 27]. A technique to carry out right hemicolectomy with a suprapubic approach and an additional extra port was described by Choo et al. and Song et al. [27, 28].

Picciariello et al. described the technique of an Abdominoperineal Resection, by placing a Uniport in the designated site for the colostomy [18].

Keller et al. have compared 50 DVSP and 50 SILS patients, who underwent various colorectal surgeries. The DVSP robotics had significantly more cases completed using only the single port and shorter LOS than SILS. The DVSP cohort also had significantly fewer cases where additional ports were needed to complete the case compared to SILS [29].

In the literature, there are 3 retrospective studies comparing DVSP versus multiport (MP) robotic anterior resection (AR) for rectal cancer. Jeong MH et al. included 13 patients per group and found that the DVSP procedures had a lower EBL and a shorter LOS, with similar docking time and operative time [30]. Complications, including the dreaded dehiscence of the anastomosis, did not differ between the two groups, 3 in DVSP and 1 MP, all treated with surgery (Clavien–Dindo 3b). Similar results are reported by Alshalawi et al. with 28 patients per arm, but with a shorter total operative time and a shorter console time in the DVSP group [31]. Kim HJ et al. found similar results in their case series of 42 DVSP vs 42 robotic multiport after propensity score matching [32] and shorter operative time, incision length, LOS, and less need for analgesics (although not statistically significant) in the DVSP group. There were no differences between the treatments in terms of complications and histopathologic outcomes.

Currently, the study with the largest case series is by Marks et al. [33]. They reported the results of a prospective phase II clinical trial of DVSP. One hundred and thirty-three patients were included: 93 (69.92%) abdominal DVSP surgeries and 40 (30.08%) transanal DVSP surgeries. The 30-day morbidity rate was 15.05 and 27.50% for abdominal and transanal cases, respectively. There was a total of three (2.65%) 30-day readmissions (two abdominal, one transanal) and no postoperative mortality. This large experience with DVSP demonstrated safety, feasibility, and favorable short-term outcomes across the spectrum of malignant and benign disease.

### Transanal surgery

The first experience of transanal robotic surgery with the DVSP was documented in a paper by Marks et al. [34]. The authors performed 12 Robotic transanal surgery in cadaveric model, subsequently another paper by Kneist et al. in 2019 documented a robotic Transanal Total Mesorectal Excision (TaTME) with the DVSP on a cadaveric model [35]; both studies demonstrated that transanal surgery with the DVSP was feasible.

In 2020, Marks et al. documented 2 cases of da Vinci single-port robotic transanal minimally invasive surgery (SP-TAMIS) for benign neoplasms, and the new system was proven to be able to offer advantages (such as camera control and dexterity with a single slim arm allowing easier access to endoluminal procedures) in comparison with Transanal endoscopic microsurgery (TEM) and TAMIS platforms [36]. Additionally, they predicted a faster learning curve due to the console being almost identical to the previous da Vinci systems. Then, the author proceeded to document the following developments in robotic transanal surgery, in particular a series of 26 SP-TAMIS [37] and a series of TaTME [38] using the DVSP. The DVSP was described as a safe and efficient system, as well as a promising tool thanks to its features of minimality and ability to work effectively in narrow spaces (Table 2).

**Table 2:** Transanal experience with da Vinci single-port robotic system

Author	Year	Procedure	Access	Intraop. complications	EBL (ml)	Post-op. compl. (C–D)	Median docking time (min)	Median operative time (min)	Positive margins (%)	LOS
Studniarek et al. [48]	2021	1TAMIS	Transanal	No	–	No	–	–	0	0
Marks et al. [45]	2021	2 TAMIS	Transanal	No	15	No	5.25	180.5	0	0.5
Marks et al. [46]	2021	26 TAMIS	Transanal	3 conversions	24.2	3 (2)	6.4	198.8	0	1.4
Marks et al. [40]	2023	40 TAMIS	Transanal	3	20	11 (< 3)	4.93	194.72	0	1
Ozgur et al. [49]	2023	10ESD	Transanal	No	–	2 (< 3b)	–	91.3	0	0

*EBL* estimated blood loss, *LOS* length of stay, *TAMIS* transanal minimally invasive surgery, *ESD* endoscopic submucosal dissection

### Cholecystectomy

Eight papers have been published concerning the initial experience with robotic single-port cholecystectomy using the DVSP on both adults and adolescents for various diseases, such as gallstone pancreatitis, symptomatic cholelithiasis, gallbladder polyps, cholecystitis, Sickle cell disease with cholelithiasis, and cholelithiasis with cholecystitis [39] (Table 3).

**Table 3:** Experience in cholecystectomy with da Vinci single-port robotic system

Author	Year	No. of cases	Access	Intraoperative complications	EBL (mL)	Post-operative complications (C–D)	Median docking time (min)	Median operative time (min)	LOS
Cruz et al. [15]	2019	1	Umbilicus	No	< 10	0	–	89	2
Cruz et al. [40]	2021	30	Umbilicus	1	11.2 ± 7.4	0	5.2	75.1 ± 17.5	1.5 ± 0.7
Kang et al. [41]	2021	72	Umbilicus	No	18.1 (13.2)	0	–	45.7 (12.2)	2.3 (0.7)
Klazura et al. [39]	2022	7	Umbilicus	No	2	0	–	126	0.28
Kim et al. [3]	2022	145	Umbilicus	No	–	9 (6 CD I, 2 CD 2, 1 CD 3B)	6.0 (5.0–8.0)	45.0 (38.0–55.0)	2.1 ± 0.8
Bianco et al. [42]	2022	141	Umbilicus	No	–	4 (CD 1–2)	2.4 (1.9)	65.5 (28.7)	0.3 (0–4)
Choi et al. [16]	2023	216	Umbilicus	2 (0.89%, (conversion)	< 50 ml	23 (CD 1–2)	3.07 ± 1.46	21.89 ± 16.10	2.39 ± 0.71
Park et al. [43]	2023	7	Umbilicus	0	–	0	–	53.6 ± 17.2	2.1 ± 0.3

*EBL* estimated blood loss, *LOS* length of stay

The first DVSP single-port robotic cholecystectomy (SPRC) was presented in 2019 by Cruz et al. [15]. The authors demonstrated such a procedure to be technically feasible and safe. In the following papers by Cruz et al. [40], Kang et al. [41], Kim et al. [3], and Choi et al. [16], SPRC has been compared to single-site robotic cholecystectomy (SSRC) with Si and/or Xi system. The SRPC has proven to be a faster procedure overall and in its subset docking time, dissection time, and console time in most of the cases and to cause the least amount of pain both on the operation day and POD 1.

This procedure was then performed by Klazura et al. [39] on 7 adolescents, two of which with sickle cell disease; this study described SPRC in adolescents as a safe and successful procedure, even in sickle cell population.

Between 2022 and 2023, two major SPRC records were published by Bianco et al. [42] and Choi et al. [16] with 141 and 216 SPRC, respectively, confirming SPRC to be a safe and feasible procedure.

Park et al. [43] presented a paper about their initial experience of gasless robotic single‐port cholecystectomy using DVSP. The unique features of DVSP enable surgeons to perform this procedure safely in an extremely reduced surgical space. The author describes gasless RSPC as a feasible and safe procedure that could help prevent the risk of CO2‐related complications due to its limited use during the procedure, lessen overall postoperative pain, and it could also eliminate shoulder pain, a typical effect of the increased intra‐abdominal pressure by CO2 pneumoperitoneum that stretches and compresses the diaphragm, in doing so the diaphragm nerve is stimulated leading to supraclavicular nerve stimulation shoulder pain.

### Pancreatic surgery

The first worldwide robotic pancreatic surgery was carried out in 2001 (and later published in 2003) by Giulianotti et al. [44]. Over 20 years of practice in this area allowed standardization of the technique. The robotic platform provides multiple and well-described benefits, especially during the pancreatic uncinate process dissection, the lymphadenectomy, and the reconstructive phase [45, 46]. Several recent studies compared robotic versus open and laparoscopic pancreatic surgery and reported similar rates of pancreatic postoperative fistula (POPF) and major complications [47, 48]. A large multicenter retrospective study also found that robotic pancreatoduodenectomy (RPD) was associated with less EBL and a shorter LOS compared with open surgery [49].

The first case of pancreatic surgery using DVSP was reported by Liu et al. in 2022. It was a distal pancreatectomy performed without surgical complications for a cystadenoma of the pancreatic tail [50].

In early 2022, Choi et al. [51] also published a miniseries of 3 patients who underwent distal pancreatectomy with DVSP with the addition of an auxiliary trocar (SP + 1) in the left flank.

Liu R et al. published their case history of pancreatic surgery using DVSP in 2022 [52]. Between late 2021 and early 2022, they performed 23 surgeries employing the SP + 1 technique (11 pancreatic enucleation, 11 distal pancreatectomy, and 1 pancreaticoduodenectomy). All surgeries were successfully performed robotically without conversion to open surgery. One patient in each of the three groups had grade B POPF and abdominal infection. No patient experienced grade C POPF or any major postoperative complications (Clavien–Dindo grade ≥ 3). No patient required reoperation or readmission to the hospital, and no 90-day mortality was observed.

Recently, Uchida et al. [53] reported the case of enucleation for pancreatic NET with the SP robot using a dual bipolar instrument (Table 4).

**Table 4:** Experience in pancreatic surgery with da Vinci single-port robotic system

Author	Year	Procedure	N	Access	Intraop. complications	EBL (ml)	Post-operative complications (C–D)	Median docking time (min)	Median operative time (min)	Positive margins (%)	LOS
Liu et al. [50]	2022	Distal pancreatectomy	1	Umbilicus	No	20	POPF-B	–	55	No	3
Choi et al. [51]	2022	Distal pancreatectomy	3	Umbilicus	No	< 500	No	4.3	215	No	10.3
Liu et al. [52]	2022	11 enucleation		Umbilicus	No	30	1 POPF-B	4.2 ± 0.8	133 ± 68.7	No	4
		11 distal pancreatectomy		Umbilicus	1 splenic v. injury	40	1 POPF-B	4.1 ± 0.5	162 ± 36.5	No	4
		1 PD		Subumb	No	200	1 POPF-B	5.8	344	No	16
Uchida et al. [53]	2024	Enucleation	1	Umbilicus	No	4	No	–	139	No	6

*EBL* estimated blood loss, *LOS* length of stay

### Upper gastrointestinal surgery

The last decade has seen an exponential diffusion of minimally invasive for Upper Gastro-Intestinal (UGI) surgery, mainly due to the growing experience of surgical teams and the availability of new technologies. In this context, the use of robotic surgery for oncologic and functional surgery of the esophagogastric tract is rapidly expanding [54]. Although laparoscopy remains the most commonly used surgical approach, robotic surgery has proven to be a safe minimally invasive approach with potential surgical advantages and excellent functional outcomes [55, 56].

Three papers concerning the initial experience with DVSP for UGI tract surgery have been published (Table 5). Two are resections for malignant pathology and one is for benign functional pathology.

**Table 5:** Experience in upper gastrointestinal surgery with da Vinci single-port robotic system

Author	Year	Procedure	N	Access	Intraop. complications	EBL (ml)	Post-operative complications (C–D)	Median docking time (min)	Median operative time (min)	Positive margins (%)	LOS
Dreifuss et al. [57]	2022	Partial Gastrectomy	1	Umbilicus	No	–	No	–	82	No	2
Cui et al. [58]	2022	Total Gastrectomy + D2	1	Subumbilicus	Subcutaneous emphysema + 7 cm incision	225	No	10	–	No	8
Cubisino et al. [17]	2022	Nissen fundoplicatio	2	left upper quadrant	No	10	No	2.5	147 (136–153)	–	2.5

*EBL* estimated blood loss, *LOS* length of stay

Dreifuss et al. [57] reported a partial gastric resection for GIST with the DVSP robot, suturing the gastric opening with a manual double-layer suture. Cui et al. [58] performed total gastrectomy with D2 lymphadenectomy for gastric adenocarcinoma. Due to the development of subcutaneous emphysema and the location of the neoplasm, they performed an extracorporeal Roux-en-Y anastomosis via a 7-cm long epigastric incision. In both cases, the postoperative course was uneventful.

Cubisino et al. described a DVSP robotic-assisted Nissen fundoplication and a redo Nissen fundoplication for gastroesophageal reflux disease, carried out safely and without complications [17].

In all the above cases, the authors report that the surgeries performed with DVSP were without complications and emphasize the feasibility of UGI surgeries with DVSP.

### Inguinal hernia repair

Minimally invasive techniques are currently accepted as the treatment of choice for primary unilateral, bilateral, and recurrent inguinal hernias (after anterior repair) due to reduced postoperative pain, wound infection, hematoma formation, and faster return to normal activities when compared to the Lichtenstein repair [1].

The unique cohort of patients was described by the Division of General, Minimally Invasive, and Robotic Surgery, University of Illinois at Chicago in 2022 and 2023 (Table 6). All surgeries were performed under an IRB-approved protocol by a single surgeon experienced in multiport robotic surgery and trained in SP robotic surgery. Dreifuss et al. [4] described the feasibility and results of SP transabdominal preperitoneal inguinal hernia repair (SP-TAPP). Bianco FM et al. compared their case series of multiport TAPP (MP-TAPP) with SP-TAPP [42]. In the two populations of 378 and 87 patients, respectively, they found significant differences in operative time (MP-TAPP: 93.2 vs. SP-TAPP: 78.1 min, *p* = 0.003) and recovery time (MP-TAPP: 160.8 vs. SP-TAPP: 112.6 min, *p* < 0.001), which was shorter in the SP group. Thirty-day morbidity, readmission rates, and chronic pain rates were similar between groups, so were hernia recurrence and port-site incision rates.

**Table 6:** Experience in inguinal hernia surgery with da Vinci single-port robotic system

Author	Year	Procedure	N	Access	Intraop. complications	EBL (ml)	30-day complications	Median docking time (min)	Median operative time (min)	LOS
Bianco et al. [42]	2022	TAPP	77	Umbilicus	No	–	5 CD < 3	2.3	79.1	0
Dreifuss et al. [4]	2023	Uni-TAPP	79	Umbilicus	No	–	7 CD < 3	2.2	78.1	0
		Bilat-TAPP	8	Umbilicus	No	–			107.2	
Lee et al. [59]	2023	TEP	1	Umbilicus	No	–	No	–	60	1

*EBL* estimated blood loss, *LOS* length of stay, *TAPP* transabdominal preperitoneal repair, *TEP* totally extraperitoneal repair, *CD* Clavien–Dindo

Lee et al. described the technique to perform a totally extraperitoneal (TEP) inguinal hernia repair using the SP Robot [59]. They demonstrated the feasibility of using an extraperitoneal approach in addition to the previously described transabdominal approach for the repair of inguinal hernia.

### Hepatic surgery

The first major series that reported the use of robotics in hepatic surgery was by Giulianotti et al. [44]. In the intervening years, liver surgery has significantly advanced thanks to continued research into minimally invasive techniques, particularly robotics, which have allowed increasingly complex procedures to be performed, optimizing patient outcomes and reducing postoperative morbidity [60, 61].

Between 2021 and 2022, Liu et al. [62] reported the first case of DVSP hepatic caudate lobectomy for hepatic hemangiomas, and Kim et al. [63] described the technique of a DVSP left lateral sectionectomy (SP-LLS) performed for intrahepatic lithiasis. In 2024, the group of Kim WJ published a study comparing 12 SP-LLS and 24 laparoscopic left lateral sectionectomy (L-LLS) after propensity score matching [64] (Table 7) Resections were mainly indicated for hepatocarcinoma. There were no differences in complications between the two groups. Compared with patients in the L-LLS group, patients in the SPR-LLS group had a longer operative time (151.8 min vs. 115.1 min, *p* = 0.004), less estimated blood loss (121.2 mL vs. 175.4 mL, *p* = 0.025) and a shorter postoperative hospital stay (5.1 days vs. 6.2 days, *p* = 0.045). They suggested that small liver resections are technically feasible and safe with the DVSP system in selected patients. A limitation identified was the lack of energy or dissection instruments specifically designed to dissect the liver parenchyma.

**Table 7:** Experience in hepatic surgery with da Vinci single-port robotic system

Author	Year	Procedure	N	Access	Intraop. complications	EBL (ml)	Post-operative complications (C–D)	Median docking time (min)	Median operative time (min)	Positive margins (%)	LOS
Kim et al. [63]	2021	Left lateral sectionectomy	1	Umbilicus	No	50	No	8	135	No	5
Liu et al. [62]	2022	Caudate lobectomy	1	Subumbilicus	No	20	No	–	–	No	2
Na et al. [64]	2024	Left lateral sectionectomy	12	Umbilicus	2 blood loss	121 ± 29	1 (2)	–	151 ± 36.5	No	5.1 ± 1.6

*EBL* estimated blood loss, *LOS* length of stay, *CD* Clavien–Dindo

### Breast surgery

The first description of robotic nipple-sparing mastectomy (rNSM) was made by Toesca et al. using a multiport robotic platform [65]. The procedure has demonstrated both acceptable safety and oncologic outcomes compared to conventional open nipple-sparing mastectomies [66, 67]. There are some technical difficulties with robotic multiport mastectomy. In particular, collisions between robotic arms, collisions between robotic and patient arms, difficulty in using the third robotic arm, and the existence of blind spots [68].

After introducing the DVSP, which aims to go beyond the difficulties mentioned, the first clinical case of rNSM with DVSP was reported by Park HS et al. and was performed in South Korea in November 2018. They performed bilateral mastectomy with immediate direct-to-implant reconstruction, which was performed by plastic surgeons without the use of the SP system [68].

The largest worldwide study using the DVSP to perform rNSM consisted of 81 cases (70 patients), for both benign and malignant pathology (Table 8). Go et al. [69] between 2018 and 2021 found no cases of conversion to conventional mastectomy and 7.5% cases of grade III complications sec. Clavien–Dindo that were comparable to what has been described in larger case histories of multiport rNSM [70].

**Table 8:** Experience in breast surgery with da Vinci single-port robotic system

Author	Year	Procedure	N	Access	Intraop. complications	EBL (ml)	Post-operative complications (C–D)	Median docking time (min)	Median operative time (min)	Positive margins (%)	LOS
Park et al. [68]	2020	Bilateral NSM	1	Anterior-midaxillary	No	No	No	5	350	No	15
Go et al. [69]	2022	Unilat NSM	59	Anterior-midaxillary	No	–	6 (3b)	–	142.5	No	–
		Bilat NSM	22					–	271	No	–
Farr et al. [71]	2024	Bilateral NSM	20	Anterior-midaxillary	No	30 (30–300)	1 (3b)	–	277 (205–351)	No	–

*EBL* estimated blood loss, *LOS* length of stay, *CD* Clavien–Dindo

Recently in 2024, Farr et al. published their case series of 20 patients who underwent SP rNSM between 2020 and 2023 in the USA with no immediate complications and histologically negative margins [71]. They note that robotic console time decreased significantly from patients 1 to 20 with no inflection point, demonstrating an optimization of the learning curve. They also found excellent results in areola-nipple complex and breast skin sensitivity, 55 and 95%, respectively, superior to larger open NSM case series [72].

### Thyroid surgery

Nine papers have been published concerning the initial experience with robotic single-port thyroidectomy using the DVSP with different approaches (Table 9). The first experience with the da Vinci SP platform was presented by Kim et al. [73]. The authors performed 10 Lobectomies with a transaxillary approach using the da Vinci SP platform demonstrating the safety of the procedure with that approach as well as an outstanding cosmetic effect owing to the shorter skin incision along the axillary skin crease combined with the reduction of operative pain or discomfort owing to the smaller extent of flap dissection reducing the risk of sensory nerve damage and minimizing paresthesia in the supra- and infra-clavicular regions (5). Kim et al. presented the first study with a large sample of 200 patients describing the outcomes of 177 lobectomies and 23 total thyroidectomies using DVSP performed by a single surgeon [74] which confirmed the previous findings of cosmetic outcomes with the added benefit of reducing the workload fatigue of surgeons. An et al. published the largest sample of patients up to date with 300 procedures including 250 less than total thyroidectomy, 31 total thyroidectomy, and 19 total thyroidectomy with modified radical neck dissection (mRND) [75]. The authors documented ease of operations provided by the easier access to deep, narrow spaces, collision-free movement, and the ease of docking, undocking, and re-docking, which is particularly advantageous during mRND where docking is performed in two stages, while maximizing the cosmetic outcomes with a reduced size of the skin incision. Moreover, these procedures did not lead to longer hospital stays as compared to other types of minimally invasive surgeries while obtaining a number of harvested lymph nodes consistent with those reported in previous studies, indicating that the dissection using SP-TART is efficient. These findings are consistent with those reported in other studies with the transaxillary presented by Ho et al. [76], Kang et al. [77], Park et al. [78] confirming that SP-TART is safe and technically feasible with a short incision length, a short hospital stay, and a relatively low complication rate with a learning curve of 20 cases for the experienced robotic surgeon as reported by Park et al. [78]. In addition to the transaxillary robotic thyroidectomy other two approaches were presented, the hairline incision by Kim et al. [79] and the areolar by Choi et al. [80]. Kim et al. improved from the unilateral or bilateral retroauricular hairline incision using the da Vinci Xi system to the unilateral incision hairline incision with the da Vinci SP thanks to the improved access to deeper and narrow spaces offered by this new platform combined with the reduced dimension of the instruments and the efficiency of the camera with its cobra mode which helped the surgeon to find the ideal view at each step of the dissection of the upper and lower poles and also the recurrent laryngeal nerve during robot-assisted thyroidectomy. Choi et al. [80] shared their initial experiences with Single-Port Robotic Areolar Approach Thyroidectomy. The authors documented an improvement in LOS, this is attributed to the marked reduction in the flap area dissection compared to the bilateral axillary breast approach (BABA) which would need bilateral axillary access instead of a unilateral as with the SP platform.

**Table 9:** Robotic thyroidectomy with da Vinci SP platform

Author	Year	No. of cases	Access	EBL (mL)	Post-operative complications	Median docking time (min)	Median operative time (min)	LOS
Kim et al. [73]	2020	10 LOB	Transaxillary	–	0	4.9 ± 1.6	148.7 ± 26.8	3
Kim et al. [74]	2022	177 LOB	Transaxillary		2	5.2 ± 1.7	116.69 ± 23.23	3.01
		23 TT				3.8 ± 2.0	176.6 ± 21.1	
						4.9 ± 1.8		
Ho et al. [76]	2022	30 LOB	Transaxillary	–	9	–	293.80 ± 36.58	4.77 ± 0.57
Kim et al. [79]	2022	32 LOB	Hairline incision	–	0	6.3 ± 3.8	119.2 ± 31.4	–
		8 IST					213.01 ± 63.1	
Kang et al. [77]	2022	81 LTT	Transaxillary	8.4 ± 5.4	10	2.1 ± 0.9	53.3 ± 13.7	2.0 ± 0.2
		16 TT		23.3 ± 48.9		2.0	86.3 ± 15.1	2.1 ± 0.3
		3 mRND		38.3 ± 53.5		4.0 ± 0.0	245.7 ± 36.7	3.7 ± 1.5
Park et al. [78]	2022	50 LOB	Transaxillary				57.8 ± 14.1	–
Choi et al. [80]	2023	17 LOB	SPRA	24.3 ± 27.3	0	–	121.7 ± 25.0	2.9 ± 0.6
		2 IST						
		2 TT						
An et al. [75]	2023	250 LTT	Transaxillary	16.2 ± 26.8	9	1.9 ± 0.8	69.8 ± 23.6	2.0 ± 0.3
		31 TT		22.8 ± 36.4	6	2.6 ± 0.7	104.2 ± 30.7	2.1 ± 0.3
		19 mRND		39.5 ± 45.7	6	5.3 ± 2.0	223.7 ± 72.4	3.1 ± 0.8

*EBL* estimated blood loss, *LOS* length of stay, *LOB* lobectomy, *TT* total thyroidectomy, *LTT* less than total thyroidectomy, *mRND* total thyroidectomy with modified radical neck dissection, *IST* isthmectomy, *SPRA* single-port robotic areolar approach thyroidectomy

## Discussion

Five years after its initial clinical report, the advantages and benefits of the DVSP robot remain uncertain compared to previous robotic surgery methods. The existing data on this platform covers various procedures, mainly in the urological field, but at the moment still lacks sufficient postoperative long-term data regarding general surgery procedures.

In the USA, DVSP has been used in urology since 2018. In a recent review, Covas Moschovas et al. reported the experience of more than 6 years, mainly for prostatectomy [81]. It was found that the DVSP is safe, and they report benefits in terms of less postoperative pain and early discharge.

Most articles in general surgery focus solely on detailing procedural techniques, surgical feasibility, and safety considerations for each approach. Furthermore, most publications suffer from small sample sizes and a dearth of long-term follow-up, hindering the possibility to evaluate functional and oncologic outcomes. These data limitations stem from the limited number of facilities that have adopted this new console. Presently, the da DVSP is only accessible in a handful of centers in the USA and in Asia, and it will begin to spread soon in Europe.

This paper discusses the initial experiences and outcomes of DVSP in various procedures in general surgery, including colorectal, cholecystectomy, pancreatic, UGI, inguinal hernia repair, hepatic, breast, and thyroid surgery.

Studies cited in this paper demonstrate the feasibility, safety, and potential advantages of DVSP over traditional laparoscopic and multiport robotic single-site approaches in terms of operative time, postoperative pain, and patient outcomes. Currently, this platform has been used in experimental protocols (not FDA approved for general surgery in the USA). Many types of procedures have been carried out, from outpatient surgery to highly complex oncologic surgery, such as hepatobiliopancreatic surgery and rectal surgery.

Dr. Marks et al. at Lankenau Institute for Medical Research and Dr Bianco et al. at the University of Illinois at Chicago have performed more than 130 major surgeries, respectively, using an SP robotic platform for colon disease and 225 between R-TAPP and RSPC and proved the safety, feasibility, and favorable short-term outcomes across the full spectrum of malignant and benign disease.

The aim of the SP approach is to reduce the surgical trauma connected to access, postoperative pain, and hospital stay and improve recovery and cosmesis. The system facilitates multi-quadrant surgery and has the potential to reduce docking time as well as set-up time [17].

Kim et al. found that in SP Hepatic Left resections, EBL and postoperative LOS were lower than MP resections [64]. Similar results were reported by Jeong et al. [30] and Kim et al. [32] in their colorectal DVSP series, and LOS and EBL were significantly lower than in MP procedures.

Regarding the cosmetic outcome, Bianco et al., after a follow-up of about nine months after 222 surgeries with DVSP, found an average satisfaction with the cosmetic outcome of 9.2 (range 4–10) [42]. This finding is consistent with a focused study published in 2021 by Morgantini et al. [82]. After administering a specific questionnaire (The Patient Scar Assessment Questionnaire) to patients undergoing DVSP and robotic multiport surgery, they reported significantly better cosmetic scar appearance and improved perception of their surgical scar in the DVSP group.

Another advantage of the DVSP is the easy-to-learn docking process which is faster than for multiport platforms. Two papers published by Choi et al. in 2022 and 2024 discuss the performance of the learning curve in colorectal SP surgery, using procedure and docking times as key metrics [83, 84]. Using a Cumulative SUM (CUSUM) curve, the authors predicted that optimal stabilization was achieved in 18 cases of colectomy and 21 cases of rectal cancer surgeries. In these cases, the mean docking time was 14.87 ± 10.38 min and 20.6 ± 19.1 min, respectively. Marks et al. found for abdominal DVSP cases that there was process control for docking after 45 cases (mean time 5.60 min, median time 4 min) [33]. Bianco et al. reported a median docking time of 2 min after 77 SP-TAPP and 141 DVSP cholecystectomies [42]. These times are shorter and more consistent than previous reports using the MP da Vinci Xi or Si robotic platforms [85].

The DVSP has a few limitations, and the technology has not yet reached its full potential. Firstly, it has no available stapler, laparoscopic stapler manipulation through the assistant port, at the SP access port can be challenging based on the task at hand. Secondly, the surgeon must rely on a trained assistant because if there is need to use advanced energy or staplers. Thirdly, the double-joined instruments tend to be in a straight position inside narrow spaces. This reduces the major advantage of robotic instruments, i.e., the angulation of the instrument tips. This is caused by the increased distance between the wrist joint and the instrument tip, requiring more space for angulating [86].

To overcome this limitation and optimize the instrument triangulation, the port cannula can be gently exteriorized or interiorized through the external chamber of SP access port with a so-called floating technique as described by Lenfant et al. [87]. The access port also allow to overcome the limitation of needing a 10-cm clearance from the entry point to obtain a full triangulation of the arms and camera. One of the main concerns and critics surrounding the robot-assisted surgery lies in its high cost of purchase and maintenance, i.e., instruments. However, the cost of any technology is an element that evolves over time and usually, the more an approach is widespread the more the cost may decrease.

Even though this issue has not been addressed for the DVSP platform yet, when considering some of its features and possible benefits may help overcome this issue.

The shorter learning curve for docking, shorter length of stay, less EBL, and shorter overall operative time may reduce the gap between laparoscopic and open surgery either optimizing the intra- and postoperative course of major procedures or fitting more outpatient procedure in one sitting while maintaining high standards of safety and effectiveness typical of the robot-assisted approach [33, 42]. A statistically significant reduction in recovery time was reported by Bianco et al. even in day surgery applications like inguinal hernia compared to multiport inguinal hernias.

A possible limitation of this technology could be the higher risk of incisional hernia formation due to the increased dimension of the incision to insert the 2.5-cm port. Bianco et al. reported a comparable incisional hernia rate after 13-month follow-up between multiport (1.1%) and single port (1.3%) robotic TAPP [4]. This technique could also play a role in faster recovery not only minimizing the traumatic effects of surgery but also thanks to the patient’s positive perception of the wound after surgery [42].

To our knowledge, this is the first systematic review to examine the experience and outcomes of the DVSP after its initial clinical use in various general surgical procedures. It is important to recognize that the existing literature is limited by the number of cases reported, the lack of long-term follow-up data, and the lack of standardized technique. In addition, the learning curve associated with this console and how it compares to other robotic systems is still under investigation. As a result, we remain committed to evaluating this technology. In future, as the system becomes more widely used, it will be possible to evaluate surgical and oncologic outcomes more rigorously through studies with larger case series and through randomized clinical trials.

The introduction of new tools, such as advanced energy devices, suction-irrigation, and staplers, may reduce the need for a bedside assistant in future while improving the number of true single-site surgeries avoiding the use of additional trocars. Overall, the DVSP platform, contrary to the previous attempts to single-site surgery, seems a promising device that, with future developments and improvements, may optimize postoperative recovery and cosmetic outcome without reducing oncologic and surgical outcomes with the concrete possibility to become a widespread platform.

## Conclusion

The experience of the last 5 years has proven that the utilization of the DVSP in general surgery procedures is both feasible and safe. Hernia repair, cholecystectomy, and colorectal surgery stand out as the most frequently performed applications. Certain groups have reported advantages, such as earlier discharge, less operative time, and reduced postoperative pain. Nonetheless, the existing literature lacks long-term follow-up data and larger patient cohorts, which are essential for substantiating the functional and oncologic outcomes linked with this platform and to compare this new technique with the other already established minimally invasive techniques.

## Supplementary Information

Below is the link to the electronic supplementary material.

Supplementary file1 (DOCX 14 KB)
