# Supplementary material for: Da Vinci single-port robotic system current application and future perspective in general surgery: A scoping review
Source: Surg Endosc. 2024 Aug 7;38(9):4814–30. doi: 10.1007/s00464-024-11126-w (PMC11362253; doi:10.1007/s00464-024-11126-w)
Supplement: Supplementary file 1 — Supplementary file1 (DOCX 14 KB) [file 464_2024_11126_MOESM1_ESM.docx]

Search: **single port AND robotic AND surgery**

("single person"[MeSH Terms] OR ("single"[All Fields] AND "person"[All Fields]) OR "single person"[All Fields] OR "single"[All Fields] OR "singles"[All Fields]) AND "port"[All Fields] AND ("robot"[All Fields] OR "robot s"[All Fields] OR "robotically"[All Fields] OR "robotics"[MeSH Terms] OR "robotics"[All Fields] OR "robotic"[All Fields] OR "robotization"[All Fields] OR "robotized"[All Fields] OR "robots"[All Fields]) AND ("surgery"[MeSH Subheading] OR "surgery"[All Fields] OR "surgical procedures, operative"[MeSH Terms] OR ("surgical"[All Fields] AND "procedures"[All Fields] AND "operative"[All Fields]) OR "operative surgical procedures"[All Fields] OR "general surgery"[MeSH Terms] OR ("general"[All Fields] AND "surgery"[All Fields]) OR "general surgery"[All Fields] OR "surgery s"[All Fields] OR "surgerys"[All Fields] OR "surgeries"[All Fields])

**Translations**

**single:** "single person"[MeSH Terms] OR ("single"[All Fields] AND "person"[All Fields]) OR "single person"[All Fields] OR "single"[All Fields] OR "singles"[All Fields]

**robotic:** "robot"[All Fields] OR "robot's"[All Fields] OR "robotically"[All Fields] OR "robotics"[MeSH Terms] OR "robotics"[All Fields] OR "robotic"[All Fields] OR "robotization"[All Fields] OR "robotized"[All Fields] OR "robots"[All Fields]

**surgery:** "surgery"[Subheading] OR "surgery"[All Fields] OR "surgical procedures, operative"[MeSH Terms] OR ("surgical"[All Fields] AND "procedures"[All Fields] AND "operative"[All Fields]) OR "operative surgical procedures"[All Fields] OR "general surgery"[MeSH Terms] OR ("general"[All Fields] AND "surgery"[All Fields]) OR "general surgery"[All Fields] OR "surgery's"[All Fields] OR "surgerys"[All Fields] OR "surgeries"[All Fields]
